# Assessing the probiotic potential, antioxidant, and antibacterial activities of oat and soy milk fermented with *Lactiplantibacillus plantarum* strains isolated from Tibetan Kefir

**DOI:** 10.3389/fmicb.2023.1265188

**Published:** 2023-09-25

**Authors:** Tariq Aziz, Hu Xingyu, Abid Sarwar, Muhammad Naveed, Muhammad Aqib Shabbir, Ayaz Ali Khan, Taqweem Ulhaq, Muhammad Shahzad, Yang Zhennai, Ashwag Shami, Manal Y. Sameeh, Sahar A. Alshareef, Manal Abdulbari Tashkandi, Rewaa S. Jalal

**Affiliations:** ^1^Key Laboratory of Geriatric Nutrition and Health of Ministry of Education, Beijing Advanced Innovation Center for Food Nutrition and Human Health, Beijing Engineering and Technology Research Center of Food Additives, Beijing Technology and Business University, Beijing, China; ^2^Department of Biotechnology, Faculty of Science and Technology, University of Central Punjab, Lahore, Pakistan; ^3^Department of Biotechnology, University of Malakand, Chakdara, Pakistan; ^4^Institute of Basic Medical Sciences, Khyber Medical University, Peshawar, Pakistan; ^5^Department of Biology, College of Science, Princess Nourah bint Abdulrahman University, Riyadh, Saudi Arabia; ^6^Chemistry Department, Faculty of Applied Sciences, Al-Leith University College, Umm Al-Qura University, Makkah, Saudi Arabia; ^7^Department of Biology, College of Science and Arts at Khulis, University of Jeddah, Jeddah, Saudi Arabia; ^8^Department of Biochemistry, College of Science, University of Jeddah, Jeddah, Saudi Arabia; ^9^Department of Biology, College of Science, University of Jeddah, Jeddah, Saudi Arabia

**Keywords:** *Lactiplantibacillus plantarum*, probiotic potential, soya milk, oat milk, antioxidant capabilities

## Abstract

Sufficient intake of probiotics has been shown to help in the digestion, protect the body against pathogenic microorganisms and boost the immune system. Recently, due to high prevalence of milk allergies and lactose intolerance in population, the non-dairy based probiotic alternative are becoming increasing popular. In this context, the oat milk and soya milk-based fermented products can be an ideal alternative for the development of Lactic acid bacteria bacteria based probiotics. These bacteria can not only improve the product’s flavor and bioavailability but also increases its antibacterial and antioxidant capabilities due to fermentation process. The purpose of the resent work was to assess the antioxidant and probiotic properties of oat and soy milk that had been fermented with three different strains of *Lactiplantibacillus plantarum* (*L. plantarum*) including *L. plantarum* 12–3, *L. plantarum* K25, and *L. plantarum* YW11 isolated from Tibetan Kefir. Different validated assays were used to evaluate the probiotic properties, adhesion and survival in the digestive system (stomach, acid and bile salts resistance), antioxidant and antimicrobial activities and safety (ABTS and DPPH scavenging assays) of these strains. Results of the study showed that soya milk and oat milk fermented with *L. plantarum* strains possess promising probiotic, antibacterial and antioxidant properties. These results can be helpful to produce dairy-free probiotic replacements, which are beneficial for those who are unable to consume dairy products due to dietary or allergic restrictions.

## 1. Introduction

Probiotics, which can be either bacteria or yeast, are “live microorganisms” that have been shown to benefit human health when consumed in adequate quantities (Rashmi and Gayathri, 2017). Probiotics are commonly made using either mono or co-culture of yeast strains like *Saccharomyces* or bacteria like *Lactococci, Lactobacillus,* or *Bifidobacterium* (Sarwar et al., 2021). The beneficial effects of probiotics on human health and well being has a long history. However, during the last three decades, a rapid increase in research on probiotic bacteria is observed across the globe. As a result, the probiotic bacteria has been extensively utilized in food industry. Mounting research evidence suggest beneficial effects of probiotics on human health. For examples, probiotics has been shown to improve digestive health, prevent the growth of harmful bacterian (Amara and Shibl,2015). They can also boost the immune (Cammarota et al., 2009) and produce of helpful cofactors and vitamins (Gu and Li, 2016), protection against cancer (Lu et al., 2021), and the reduction and prevention of high blood pressure (Kerry et al., 2018).

Different criteria are used to assess the desirable features of potential probiotic strains that is suited for human and animal usage. Of these, the capacity of the probiotic bacteria to colonize the host’s digestive system is one of the most crucial factor. This happen when bacteria of the same strain aggregate together, as in bacterial clumping, or when bacteria of different strains cluster together, as in coaggregation. As a result of adhesion, the probiotic bacteria can activate the immune response and prevent pathogenic bacterial by competitive (Dlamini et al., 2019). Antioxidant activity is another important consideration of probiotics. Degenerative illnesses, including cancer, Alzheimer’s, and Parkinson’s, may be caused by oxidative damage, and antioxidants play a crucial role in preventing this damage. Antioxidants play a role in important cellular processes like cell signaling, gene expression, apoptosis, and ion transportation by delaying or preventing the oxidation of cellular substrates targeted by reactive oxygen species (ROS) like superoxide anion (O2−), hydrogen peroxide (H_2_O_2_), and hydroxyl radical (−OH) (Lü et al., 2010). Safety of probiotics is also an important aspect of probiotics use which is commonly assessed through safety assessment studies including antibiotic resistance test, hemolysis test, and the presence of virulence factor genes (VF). Horizontal transmission of such elements to the host’s native flora or to pathogenic bacteria that are only there momentarily is theoretically possible (Klibi et al., 2013).

Moreover, the lactic acid bacteria (LAB) are known to produce various short-chain fatty acids (SCFAs) that contribute significantly to innate immunity. Among the SCFAs, propionic acid, acetic acid, butyric acid, isovaleric acid, isobutyric acid, and valeric acid hold particular importance due to their anti-inflammatory properties and their role in regulating the gut microbiota. These SCFAs play a vital role in maintaining a balanced and healthy digestive tract, ensuring smooth functioning and overall well-being (Kang et al., 2021).

The useful effects of lactic acid bacteria (LAB) have led to their increased popularity. The FDA has approved LAB as a safe probiotic for use in food and animal feed primarily due to its capacity to attach to the gastrointestinal mucose, inhibit pathogen colonization and growth and proven antioxidant activity. Moreover, LAB bacteria can inhibit inflammation and regulators of the gut microbiota through short-chain fatty acids (SCFAs) production by LAB, Beneficial effects, such as cholesterol reduction and antioxidant activity, have been seen in LAB strains isolated from fermented dairy products, making them a valuable bioresource for the discovery of probiotic bacteria with functional qualities (Ismael et al., 2022).

Due to its high protein and phytochemical content, soymilk is a viable dairy milk alternative for the consumers. Soymilk contain bioactive phenolic compounds which have been linked to a lower the risk of cardiovascular issues and cancers (Rizzo and Baroni, 2018). Since it contains all the necessary amino acids, soymilk is also a suitable alternative for those who are lactose intolerant or allergic to milk proteins. Soymilk has many health benefits, but its beany taste and gas-inducing properties has lowered its chances to become the frontline milk alternative. Fortunately, fermentation process has been shown to overcome the problems associated with soy milk consumption especially the beany flavor which is greatly diminished by fermentation thus making it more palatable to consumers. Fermentation also reduces the flatulence-inducing oligosaccharides, including verbascose, stachyose, and raffinose to a greater extent. Moreover, Soymilk’s bioavailability of vital elements such as minerals, vitamins, protein, and isoflavones is enhanced through fermentation. The ultimate results is improved health benefits of soymilk and by fermenting it with probiotic bacteria, can pave the way for the development of new soy-based products that may satisfy a wider variety of customer tastes. This would surely add to enhance taste and palatability thus posing a great alternatives to regular milk (Battistini et al., 2018). Another important benefit of fermented soymilk due to β-galactosidase activity which catalyzes the conversion of isoflavone glycosides into aglycones. Aglycones are the bioactive form of isoflavones and responsible for their beneficial effects especially cancer prevention (Sánchez-Calvo et al., 2013).

Probiotic strains have demonstrated history of enhanced survival in fermented dairy foods and hence these foods have been employed for a long time as a vehicle for delivering probiotics. However, the ever increasing demand for dairy free probiotic meals that do not include dairy due to issues such as lactose intolerance, milk allergies, and the rising popularity of low-cholesterol options, the search for alternative products is on the rise. Probiotic fermented non-dairy products’ benefits include lactose-free and having a lower cholesterol level than dairy-based alternatives. There has been a rise in the popularity of fermented cereal, fruit, and vegetable products that include probiotic bacteria in recent years. There are a number of reasons why people are interested in these substitutes for dairy products. First, they provide alternatives for those who wants dairy-free products due any medical or psychological reason. Second, the complex carbohydrates included in many of these products are a favorite food of probiotics bacteria that enhances their activities in the intestines. There is a wide variety of nutritional and allergy-friendly non-dairy probiotic alternatives thanks to the inclusion of grains, fruits, and vegetables in this formulation (Chen et al., 2020).

Cereals are suitable for producing a wide range of food compositions that may give health advantages because they provide an ideal environment for the growth of lactic acid bacteria (LAB) with probiotic potential (Salazar et al., 2009). Non-digestible dietary fibers, such as oat β-glucan, have attracted interest in the development of functional foods owing to their many valuable and beneficial qualities. Insulin resistance, dyslipidemia, obesity, hypertension, and cancer are just a few of the disorders that oat β-glucan has been shown to improve. Products made from fermented oats, including yogurt substitutes, have been produced with extra β-glucan thus enhancing its health benefits. Fermented oat drinks that include probiotic LAB have also improved the functional potential of oat beverages (Angelov et al., 2006). Oats have also been used as a functional component to improve the effectiveness of non-dairy probiotic blends. Recent developments in biotechnology have made it possible to use riboflavin overproducing strains of *Lactiplantibacillus plantarum* and *Limosilactobacillus fermentum* to significantly boost the vitamin B2 content in cereal-based fermented foods (Capozzi et al., 2012). The development of functional cereals using LAB strains with the ability to produce B-group vitamins has shown encouraging results. Some LAB strains have the unique capacity to synthesize exopolysaccharides (EPS), which is another interesting property of microbial food cultures. LAB-produced EPS are fermentable prebiotic compounds that can modulate the gut microbiota, making them useful molecules in functional foods (Salazar et al., 2016). The goal of this study is to determine whether Tibetan kefir-derived *Lactiplantibacillus plantarum* strains have any potential for fermenting oat and soy milk. The study seeks to examine the viability and probiotic properties of the strains, measure antioxidant activities and compare their genomic properties. To comprehend microbial interactions, microbial ecology were studied by state of the art genetic tools. The study’s results may reveal brand-new functional foods with improved probiotic and antioxidant capabilities, advancing knowledge of their potential health advantages.

## 2. Materials and methods

### 2.1. Sample collection and proximate analysis

The samples of oat and soymilk were purchased from the local market in Beijing China, transported in sterile containers, and stored at 4°C for further investigation. The samples’ electric conductivity (EC) and pH levels were determined using a digital pH meter. Moisture analyzer was used to determine moisture contents in each sample. In order to assess the ash contents, five grams of the samples were heated in an electric muffle furnace for at least five hours and temperatures range between 550 and 600°C. Utilizing the Kjeldahl nitrogen technique, the amount of crude protein in the fresh milk samples was also determined. Total unrefined fat content was calculated using the Soxhlet apparatus.

### 2.2. Isolation and characterization of *Lactiplantibacillus plantarum* strains from fermented food samples

To isolate LAB strains, 0.5 g of the samples were crushed using a mortar and mixed with 4.5 mL of a 0.85% (w/v) saline solution. The resultant mixture was further serially diluted in 0.85% (w/v) saline and subsequently spread onto de Man, Rogosa, and Sharpe (MRS) agar plates and placed in an anaerobic incubator (YY-S, Maworde, China) at 37°C for a period of two to three days. Following incubation, individual colonies were isolated from the plates and cultured in a liquid MRS medium under the same temperature and humidity conditions. The collected strains were initially examined using a microscope to determine their identities. To further identify the strains, 16S rDNA sequencing was performed. Among the isolated LAB strains, three *L. plantarum* strains (YW11, 12–3, and K25) were selected and stored in a 40% (v/v) glycerol stock at −80°C for subsequent investigations.

#### 2.2.1. Whole genome analysis and genome characterization

The current study utilized EZbioCloud platform^1^ for whole genome analysis and genomic characterization of *Lactiplantibacillus plantarum* strains YW11, 12–3, and K25. The genomic sequences of *Lactiplantibacillus plantarum* strains YW11, 12–3, and K25 were obtained in FASTA format from public databases. These sequences were subjected to quality control checks to assure accuracy and dependability. Various bioinformatics tools on the platform were used to assess genomic properties such as gene content, gene organization, and structural changes.

### 2.3. Pan-genome analysis

The EZbioCloud platform was used to undertake a pan-genome analysis of *Lactiplantibacillus plantarum* strains YW11, 12–3, and K25. To begin, public databases were used to get the genomic sequences of *Lactiplantibacillus plantarum* strains YW11, 12–3, and K25. These sequences were obtained in FASTA format and were subjected to quality control procedures to verify accuracy and dependability. The EZbioCloud platform, which provides a comprehensive set of bioinformatics tools for genome study, was then used. The Pan Genome study Pipeline, which is available on the platform, was used to execute the pan-genome study. This pipeline identifies core genes, accessory genes, and strain-specific genes within the pan-genome using a combination of computational techniques and statistical methodologies.

### 2.4. Venn diagram

A Venn diagram was used to assess the functional distribution of *Lactiplantibacillus plantarum* strains YW11, 12–3, and K25, providing insights into the strains’ common and unique functional categories. The Venn diagram illustrated the intersection and exclusive zones of functional categories ascribed to each strain’s genes.

### 2.5. Assessment of the probiotic properties of *Lactiplantibacillus plantarum* strains

#### 2.5.1. Acid and bile salt resistance

We further assessed the acid and bile salt tolerance of different *Lactiplantibacillus plantarum* strains. For this purpose, MRS (de Man, Rogosa, and Sharpe) media was prepared with different concentrations of bile salts (ranging from 0.10 to 0.50%, w/v) and pH (5.80, 5.10, 4.40, 3.60, and 3.00) and inoculated with an overnight cultures of *L. plantarum* strains. The cell suspensions, with an initial optical density (OD) of 0.10 ± 0.05 at 600 nm (OD600), were then dispensed into the wells of 96-well plates. The plates were then sealed and incubated in a log-phase 600 incubator at 37°C and 500 rpm for a duration ranging from 24 to 96 h. MRS media and cell inoculum were used as negative controls. Throughout the incubation period, the optical density of the culture (OD600 nm) was recorded at regular 10-min intervals. The strains’ ability to withstand acid and bile salt was assessed by measuring the time it took to enter the lag phase, representing the time needed to adjust to the inhibiting circumstances. Each experiment was conducted at three different time points. Tolerance was calculated by comparing the survival rates of cultures incubated in MRS broth containing bile salt to those incubated in MRS broth without bile salt (the control group).

The following equation (Eq. 1) was used to determine the tolerance percentage:


(1) $$Bileresistance\;\left(\%\right)=\frac{\left(A\left(t\right)620nm\right)}{\left(A\left(0\right)620nm\right)}\times 100$$

where A(t) is the culture’s growth in bile salt-supplemented MRS broth and A(0) is the culture’s growth in bile salt-free MRS broth.

### 2.6. Adhesion test

The hydrophobicity and auto-aggregation index of the strains were measured in accordance with the standards set out by Lee and Kim in 2019 (Lee and Kim, 2019). In order to get under adhesion properties of the strains, these two indices were assessed. Triplicates of all experiments were conducted for statistical reliability. At the same time, 3 mL of a cell surface hydrophobicity (ICS) indicator was combined with 1 mL of xylene to determine hydrophobicity. Phase separation, which resulted in an upper and lower phase, was induced by incubating the mixture at 37°C. The hydrophobic fraction, which is found in the bottom phase, was extracted for further study. The absorbance of the collected lower phase at 600 nm, designated as At, was used to calculate the hydrophobicity of the strains. This equation (Eq. 1) was used to determine the hydrophobicity:


(2) $$Hydrophobicity\;\left(\%\right)=\left(1-\frac{At}{A0}\right)\times 100\;$$

In this case, A0 is the samples’ initial OD600 nm at *t* = 0 h.

Auto-aggregation was measured by incubating 4 mL of ICS at 20°C for 24 h. Then, the OD600 nm of 1 mL of the collected upper layer solution was determined. Equation (2) was used to find the auto-aggregation index:


(3) $$Auto-aggregation\;\left(\%\right)=\left(1-\frac{A24}{A0}\right)\times 100\;$$

In this formula, A24 is the top layer’s OD600 nm at *t* = 24 h, and A0 is the starting OD600 nm at *t* = 0 h. By taking these readings, we were able to put a number on the auto-aggregation seen in the strains. These techniques allowed us to assess the strains’ hydrophobicity and auto-aggregation properties, which shed light on their adhesion potential.

### 2.7. Survival in simulated human gastrointestinal transit

To further investigate how well different strains of *Lactobacillus plantarum* may survive in digestive system, a simulated gastric juice (SGJ) and simulated small intestinal juice (SSIJ) was developed to mimic the digestive fluids produced in the stomach and small intestine, respectively. The SGJ comprised a saline solution with a pH of 2.50 and a concentration of 27 mg/mL of pepsin (Solarbio, Beijing, China). The SSIJ was prepared using pancreatin (Solarbio, Beijing, China) solution at 0.27 mg/mL in a 0.50 percent saline solution at pH 8.00. The cells of *L. plantarum* strains that had already been cultured were collected and suspended in SGJ at a concentration of 10^8^ CFU/mL and incubated at 37°C for three hours. After incubation, 0.5 mL of the SGJ samples were mixed with 4.5 mL of the SSIJ to mimic the environment of the small intestine and the mixture was further incubated at 37°C for an additional 3 h.

Cells survival was assessed by inoculating MRS agar plates with the cultures acquired from the simulated gastrointestinal transit tests and counting viable cells as described by Fei et al. (2018). Each experiment was repeated three time to ensure accurate and consistent results.

The following equation was used to determine the survival of the strains in simulated gastric and small intestine environments.


(4) $$SurvivalRate\;\left(\%\right)=\frac{\begin{array}{l} TotalNumberofViable\\ Cells\;on\;MRS\;AgarPlate \end{array}}{InitialCellDensity\;}\times 100\;$$

### 2.8. Antioxidant activity

The antioxidant properties of the different *Lactiplantibacillus plantarum* was assessed using 2,2-diphenyl-1-picrylhydrazyl (DPPH) radical scavenging activity assay and the 2,2′-azino-bis(3-ethylbenzthiazoline-6-sulfonic acid) (ABTS) radical cation scavenging activity following the procedures laid out by Tang et al. For the DPPH radical scavenging activity experiment, 0.50 mL of the sample (ICS) or 0.50 mL of a 0.20 mmol/L DPPH solution were combined. The control was 0.50 mL of phosphate-buffered saline (PBS). The DPPH solution was replaced with methanol to serve as a blank. The resulting mixes were left to incubate for 30 min at 37°C in the dark. The mixes were then centrifuged for 10 min at 12,000 rpm. Microplate reader (M5, SpectraMax, USA) readings for absorbance at 517 nm were taken from the resultant solutions. The antioxidant activity was determined by measuring the decrease in absorbance induced by the DPPH radical being scavenged by the strains.


(5) $$\begin{array}{l} DPPHRadical\\ ScavengingActivity\;\left(\%\right)=\left(1-\frac{\left(A\;sample-A\;blank\right)}{\;A\;control}\right)\;100 \end{array}$$

To perform the ABTS radical cation scavenging experiment, we mixed 7.40 mmol/L of ABTS+ with 40 mmol/L of potassium persulfate in a working solution. This mixture was given 12 h to react in the dark at room temperature. After reaching an absorbance of 0.70 0.02 at 734 nm, the working solution was diluted with 100% ethanol and kept at 30°C for later use. Sample (ICS) or control (phosphate-buffered saline, PBS) volume of 0.25 mL was combined with ABTS working solution volume of 4 mL to perform the experiment. The working solution was substituted with 4 mL of 100% ethanol for the blank samples. After that, we incubated all of the samples at 30°Cfor 6 min. After the samples had been incubated, their absorbance was compared to that of blank samples at 734 nm. Absorbance decreases were used to determine sample performance in the ABTS radical cation scavenging experiment. Antioxidant activity against the ABTS radical cation was calculated by comparing sample absorbance to that of a blank.


(6) $$\begin{array}{l} ABTSRadical\\ ScavengingActivity\;\left(\%\right)=\left(1-\frac{\left(A\;sample-A\;blank\right)}{A\;control}\right)\;100\;\; \end{array}$$

### 2.9. Antibacterial activity

The agar diffusion test was used to determine how well the chosen strains inhibited the growth of bacteria, based on a procedure reported by Wang et al. (2018). This assay is commonly used to determine whether or not an antibiotic/antibacterial agent can stop the growth of bacteria. Luria Bertani (LB) agar plates with 7 mm wells were prepared, and 100 L of cell-free supernatant (CFS) from the test strains was carefully added to each well. Indicator microorganisms, including *Staphylococcus aureus*, *Escherichia coli*, *Shigella flexneri*, and *Salmonella typhimurium* were previously dispersed on these LB agar plates. Indicator bacteria are used to determine the effectiveness of an inhibiting environment in promoting the development of the target strains.

The plates were then incubated at either 30°C or 37°C for 48 h, depending on the ideal growth conditions for the indicator bacteria, after which the CFS was added to the wells and allowed to dispersed into the surrounding agar medium. An inhibitory zone surrounding the wells showed that the strains had antibacterial activity. The amount of antibacterial activity was determined by measuring the diameter of the inhibitory zone from one edge to the other. The stronger antibacterial potential is indicated by a wider width of the inhibitory zone (Liu et al., 2020; Sethi and Tao, 2022).

### 2.10. Hemolytic activity

The hemolytic activity of the strains was assessed by streaking a loopful of pre-culture test strains onto Columbia blood agar plates with 5% sheep blood (Sharma et al., 2018). Hemolytic activity was detected by checking for the presence or absence of translucent rings around the colonies. If no such rings formed, then the strains may be safely used as probiotics since they lacked hemolytic activity.

### 2.11. Bile salt hydrolase activity

The capacity of a strain to break down bile salts is measured by its bile salt hydrolase (BSH) activity. For this purpose, a stomach-like environment was created on MRS agar plates by adding 0.37 g/L CaCl2 and 5 g/L sodium taurocholate. The intracellular supernatant (ICS), or 100 μL of the cell suspension, was put to 7 mm wells on MRS agar plates to measure the bile salt hydrolase (BSH) activity of the various strains. The plates were placed in an incubator after the inoculated cell suspensions (ICSs) were applied to the wells. During the incubation phase, any strain with BSH activity was detected by precipitation or turbidity in the wells. This turbidity or precipitation showed that the strain could hydrolyze the bile salts in the agar media.

### 2.12. Statistical analysis

The IBM SPSS Statistics 26 software was used to conduct statistical analysis. The data were presented as the mean and standard deviation (SD) calculated from three independent replicates. Using Duncan’s Multiple Range Test, calculations were performed with a significance difference of *p* < 0.05 to identify significant differences between the parameter means.

## 3. Results

### 3.1. Whole genome analysis and genome characterization

**Figure 1:**
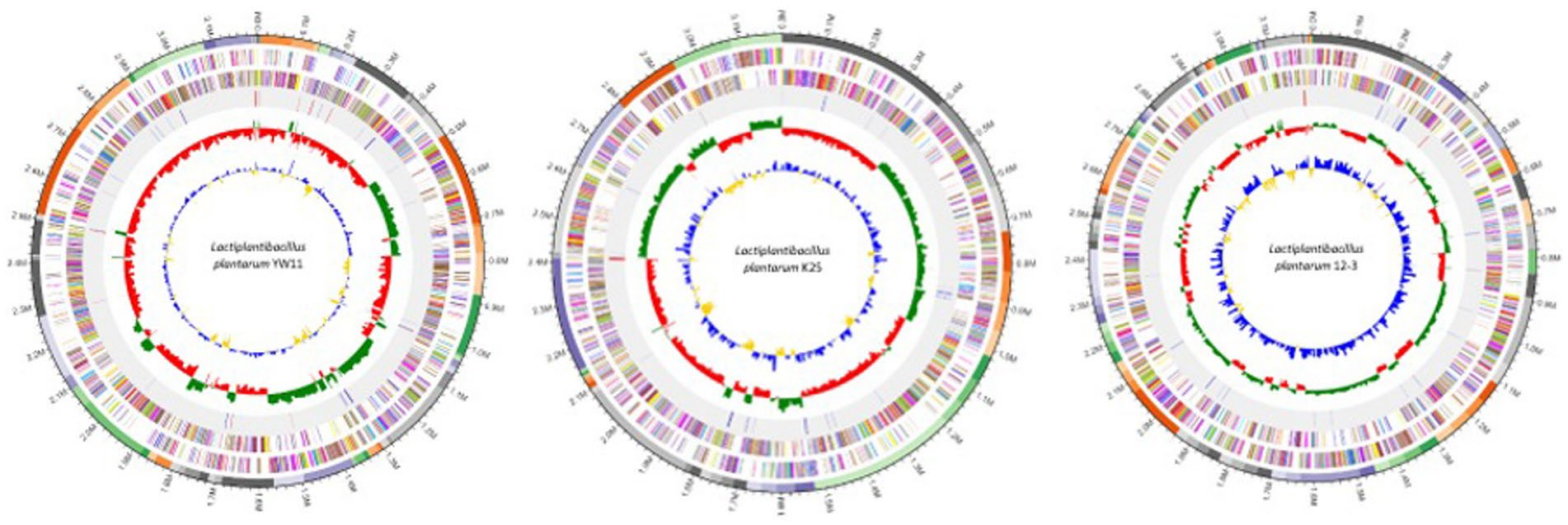
Genomic maps of *Lactiplaantibacillus plantarum* YW11, K25, and 12–3.

The genomic characteristics analysis revealed the genome sizes of the three strains ranged from about 2.8 to 3.2 megabases, with equal GC content across all strains. Repeated elements, such as transposable elements and insertion sequences, were found in different strains, indicating possible genomic flexibility. It was observed that the strains had distinct functional profiles after identifying virulence factors, antibiotic resistance genes, and other functional features. The presence of these components differed among strains, indicating potential changes in virulence potential and antibiotic resistance. The comparative genomics study revealed both shared and distinct genetic characteristics. The examination of orthologous genes identified a core collection of genes that were found in all three strains, demonstrating conserved activities and critical biological processes. Furthermore, strain-specific gene clusters related with metabolic pathways and functional properties were discovered, emphasizing the genetic diversity and functional variability found within the *L. plantarum* species. The genome maps of the all three genomes are presented in the Figure 1.

### 3.2. Pan-genome analysis

**Figure 2:**
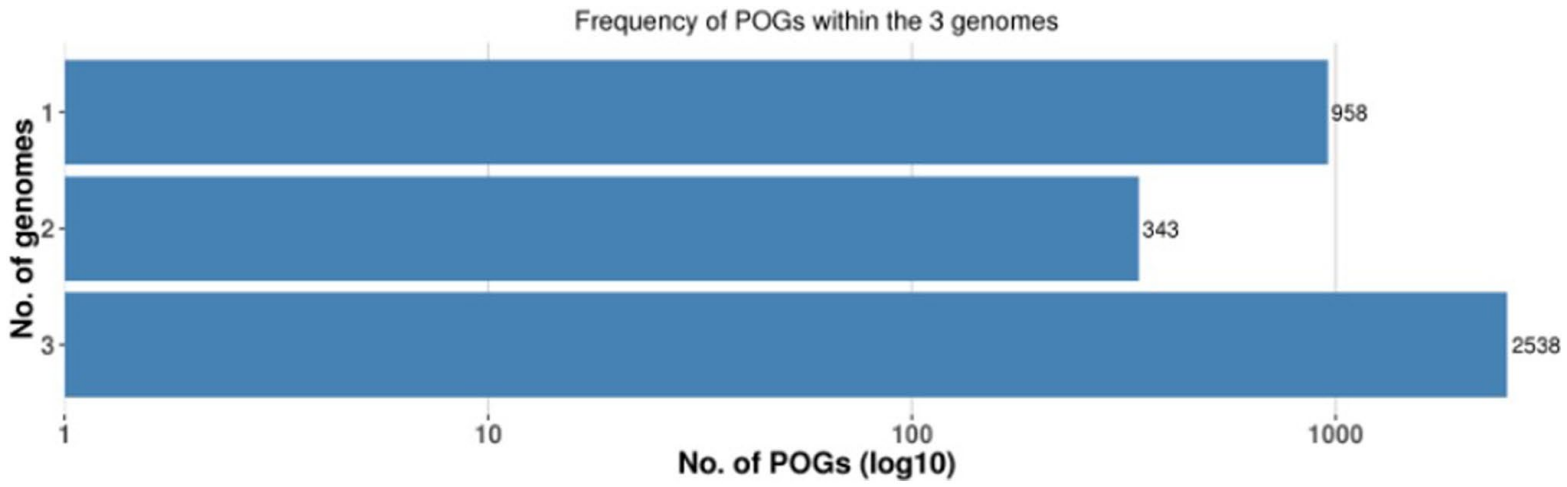
Frequency plot of POGs within the 3 genomes.

**Figure 3:**
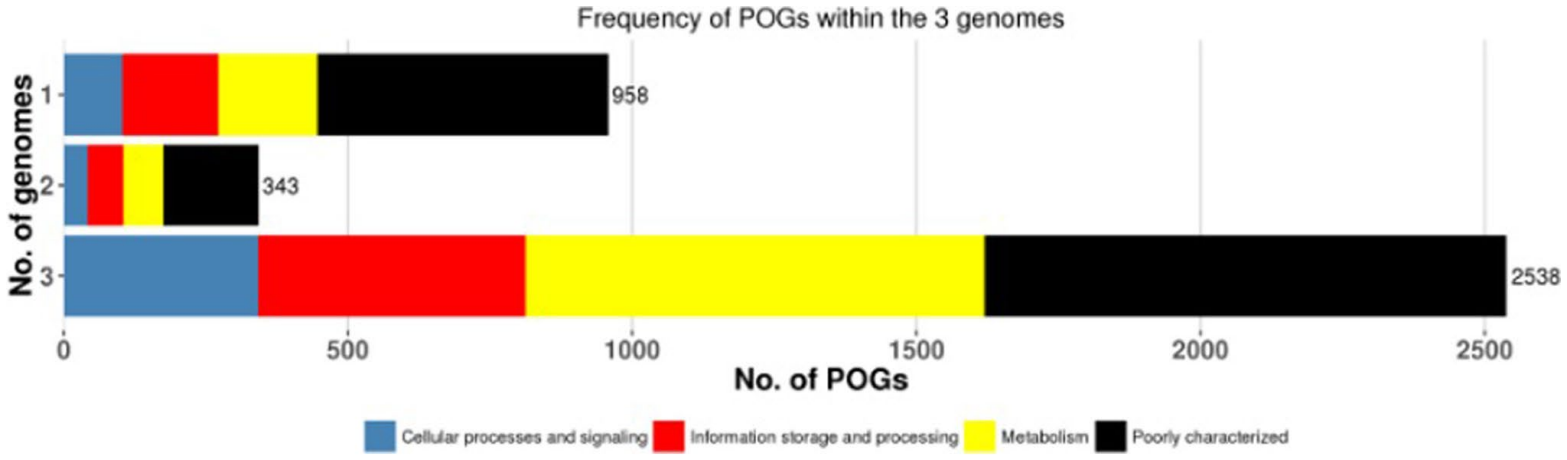
The frequency plot of the 3 genomes with major COG categories.

A pan-genome frequency plot was created to show the distribution of core genes, accessory genes, and strain-specific genes. The frequency plot also revealed information on the genetic flexibility of *L. plantarum* strains. It showed the existence of a large number of accessory genes, implying that these strains had a highly flexible pan-genome capable of gaining additional genetic elements via horizontal gene transfer or gene loss. The frequency plot of the POGs within 3 genomes is given in the Figure 2 and the frequency plot with the major COG categories is given in the Figure 3.

**Figure 4:**
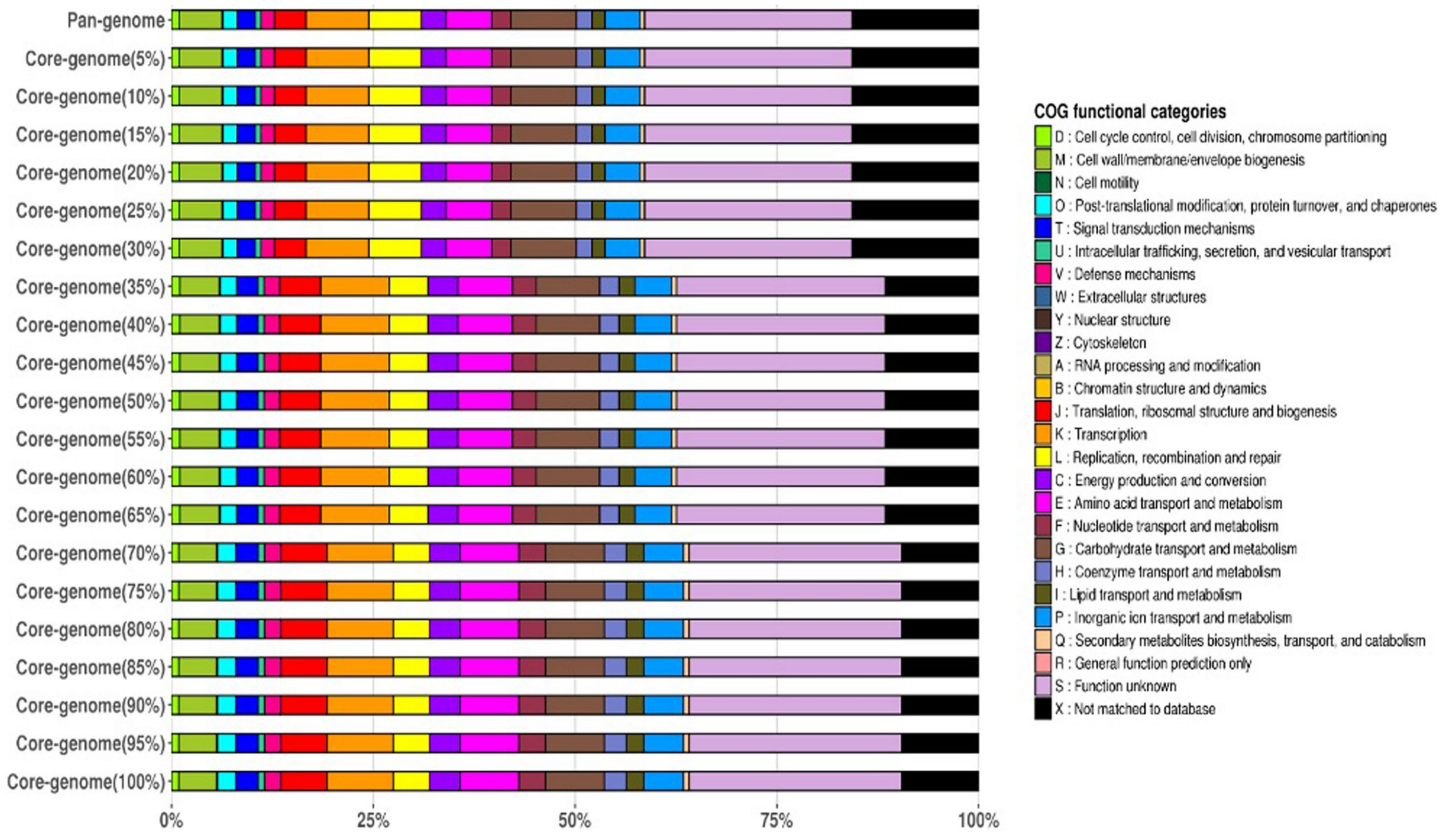
Functional categories of the genomes by COG/EggNoc Scheme.

**Figure 5:**
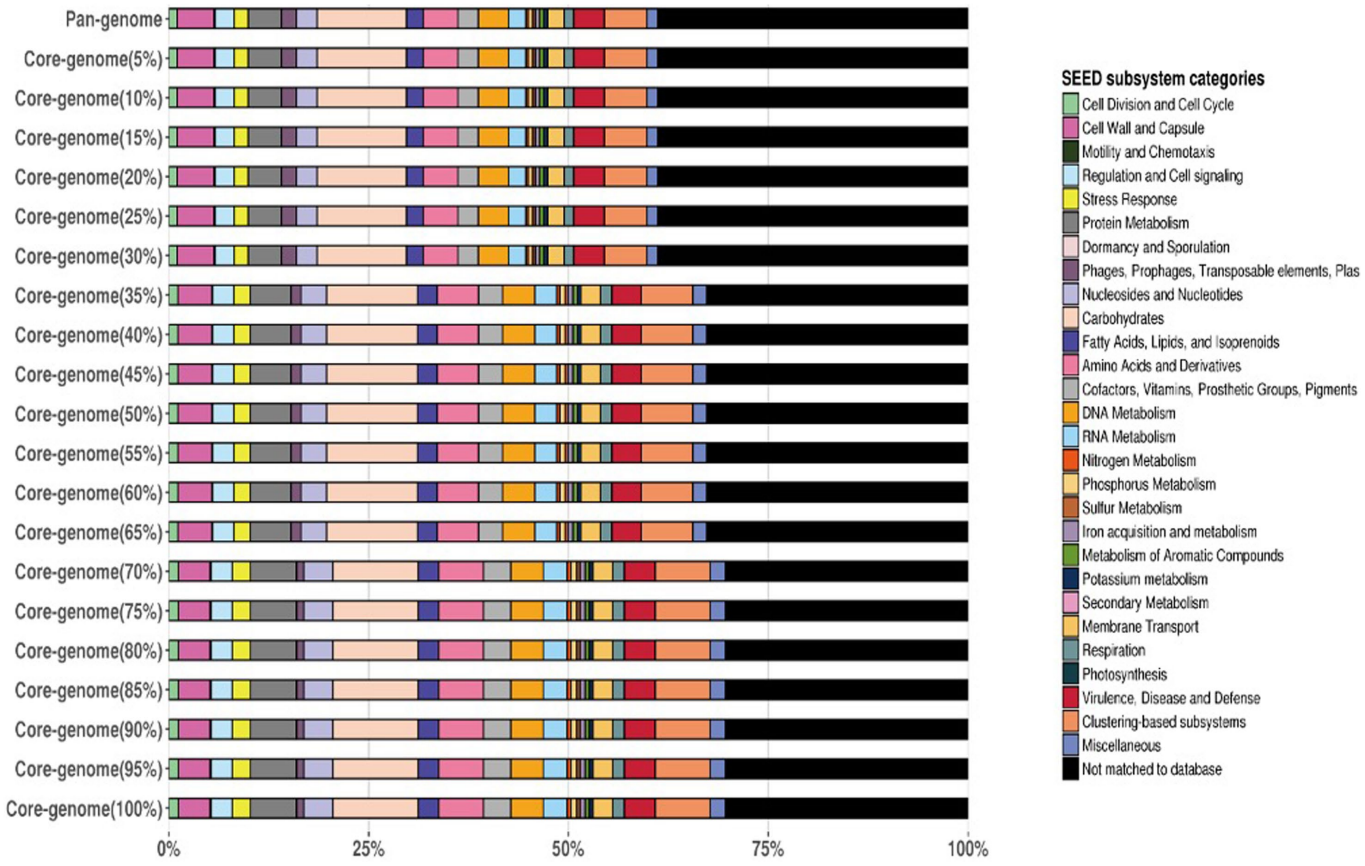
Functional categories of the genomes by SEED Scheme.

The frequency plot revealed that the *L. plantarum* pan-genome consisted of a core set of genes shared by all three strains, as well as accessory genes present in some but not all strains. The core genes represented the conserved genomic elements required for the species’ basic biological processes, whereas the accessory genes revealed genetic variants that contributed to strain-specific traits. The frequency plot, however, revealed a significant number of strain-specific genes, showing the presence of distinct genetic characteristics in each strain. These strain-specific genes could be important in conferring various phenotypic features or adaptations to specific settings. Figure 4 depicts the functional categories of the genomes by COG/EggNogg Scheme and the functional categories by SEED scheme are given in Figure 5.

### 3.3. Ven-diagram

**Figure 6:**
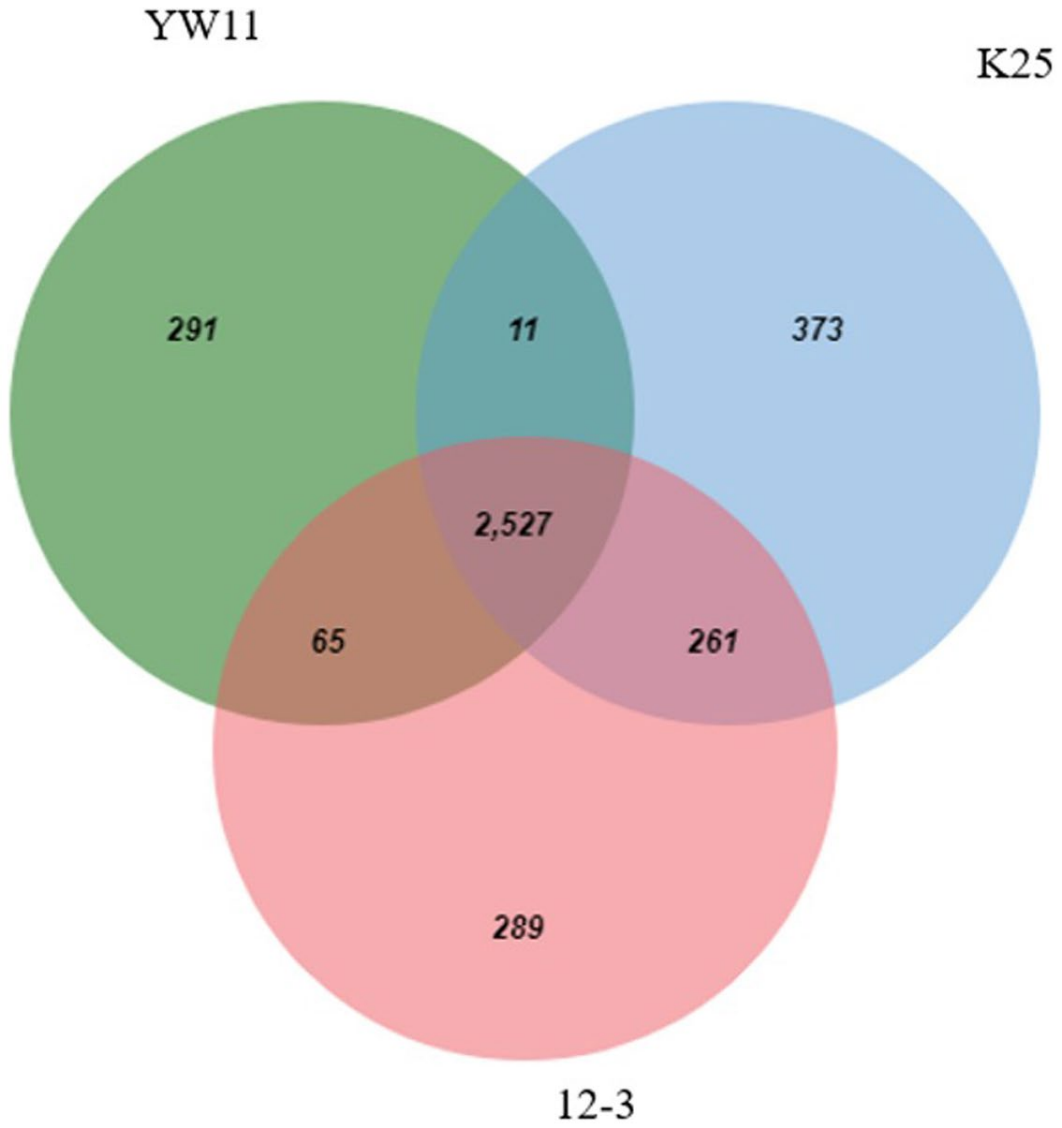
Venn-Diagram depicting the functional genes in all 3 genomes.

Considerable overlap in functional categories across the strains was observed indicating that all three strains share a core set of tasks. These shared functional categories are most likely critical biological activities and conserved metabolic pathways required for *L. plantarum* survival and proliferation. Furthermore, the Venn diagram revealed different functional categories that were only found in one strain, showing the presence of strain-specific functionalities. Individual strains’ unique functional categories may represent specific adaptations or specialized metabolic capabilities. Strain YW11, for example, had a distinct functional category linked with probiotic-related functions, indicating its potential for probiotic uses. The Venn diagram is given in the Figure 6.

### 3.4. Proximate analysis

The proximate analysis of oat milk and soy milk is presented in Table 1. These data show that oat milk and soy milk are chemically unique, having different amounts of ions, acids, minerals, proteins, fats, and water as shown in Table 1.

**Table 1:** The proximate analysis of oat milk.

Strain	Electric Conductivity (ms/cm)	pH	Ash Content (%)	Protein (%)	Crude Fat (%)	Moisture (%)
Oat Milk	2,257	6.14 ± 0.02	0.78 ± 0.07	4.02 ± 0.4	3.27 ± 0.08	87.67 ± 0.38
Soya Milk	2,587	6.28 ± 0.1	0.68 ± 0.04	3.98 ± 0.3	3.56 ± 0.02	84.89 ± 0.0.40

### 3.5. Acid and bile resistance

All the three tested strains showed remarkable resistance to both bile salts and acidic environments. The strains required more time to adjust to the restrictive environment as the pH dropped and the concentration of bile salts rise. *Lactiplantibacillus plantarum* YW11 was the most resistant to bile salts and acids, with the shortest lag phase periods and greatest bile resistance percentages across the board. In contrast *L. plantarum* 12–3 and *L. plantarum* K25 strain possess comparatively less acid and bile salt-resistant as shown in Table 2.

**Table 2:** Adaptation of three strains of *Lactiplantibacillus plantarum* to gastrointestinal environment through measuring lag phase growth in different concentrations of bile resistance.

S. No	Strain types	pH	Bile concentration (%)	Time h	Bile resistance (%) ± SD
1	*L. plantarum YW11*	5.7	0.1	4.5	77.53 ± .94^a^
			0.2	5.2	84.40 ± .62^b^
			0.3	5.8	89.80 ± 1.44^c^
			0.4	6.4	92.50 ± 1.11^d^
			0.5	7.1	97.16 ± .602^e^
2	*L. plantarum 12–3*	5	0.1	6.1	84.33 ± .96^a^
			0.2	7	88.83 ± .95^b^
			0.3	7.8	93.93 ± 1.32^c^
			0.4	8.6	96.90 ± 1.47^d^
			0.5	9.3	99.43 ± .35^e^
3	*L. plantarum K25*	4.3	0.1	6.6	85.53 ± 1.49^a^
			0.2	7.6	91.87 ± 1.63^b^
			0.3	8.3	95.36 ± .86^c^
			0.4	9	97.53 ± 1.07^cd^
			0.5	9.7	99.13 ± .73^d^

Every value is the mean of three replicates ± shows standard deviation [SD] among the replicates. The values of the columns are compared statistically. The various small alphabetic letters (abc) in respective column significantly differs from each other.

### 3.6. Adhesion ability

**Figure 7:**
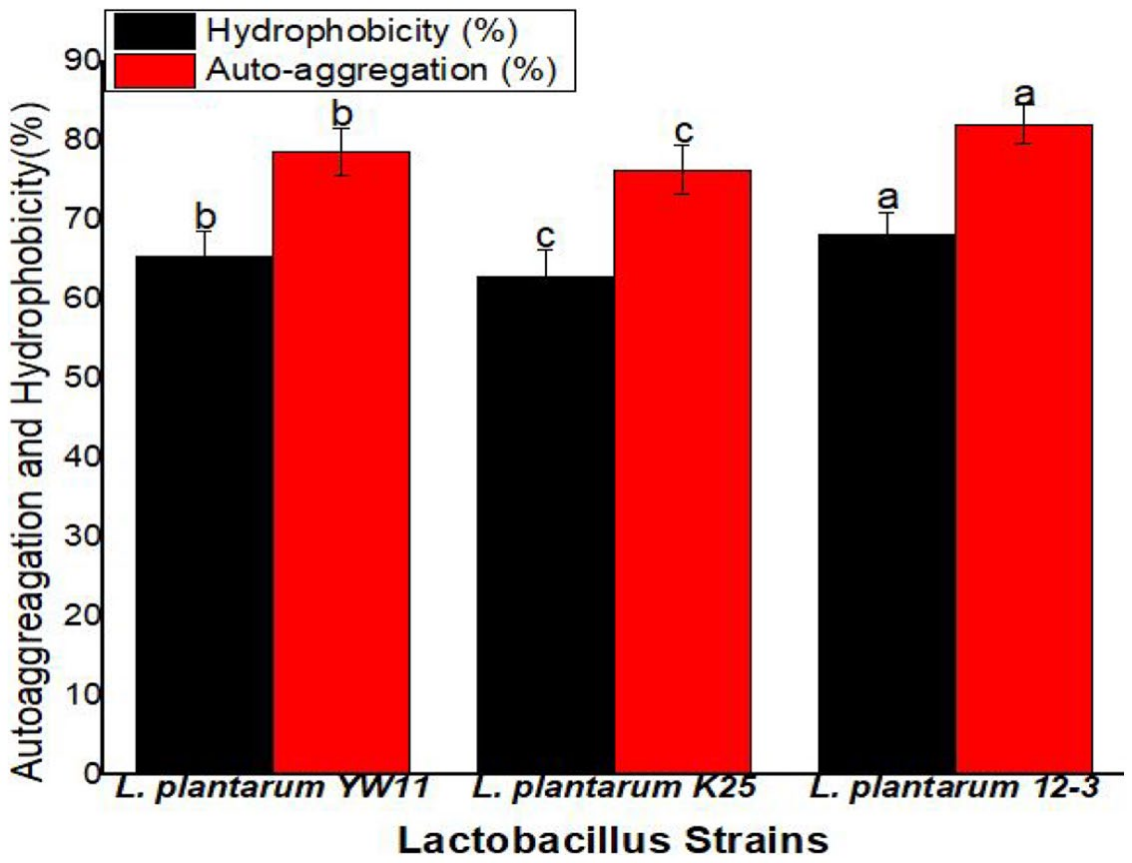
The adhesion ability of three strains of *L. plantarum* through Hydrophobicity and Auto-aggression test. The letters a, b, c showing significant differences (*p* < 0.05) within each column.

Hydrophobicity values across the strains ranged from 68.1 ± 2.8% for *L. plantarum* 12–3 to 65.4 ± 3.2% for *L. plantarum* YW11 and 62.8 ± 3.5% for *L. plantarum* K25. Auto-aggregation was greatest for *L. plantarum* 12–3 (82.1 ± 2.5%) followed by *L. plantarum* YW11 (78.6 ± 2.9%), and *L. plantarum* K25 (76.3 ± 3.1%). All three strains have remarkable adhesion capabilities, with *L. plantarum* 12–3 demonstrating the greatest adhesion potential with regard to of hydrophobicity and auto-aggregation, according to the findings. These strains’ probiotic advantages are backed up by their hydrophobicity and auto-aggregation characteristics, which allow them to adhere to surfaces in the gastrointestinal tract (Figure 7).

### 3.7. Survival rate in simulated human gastrointestinal transit

**Figure 8:**
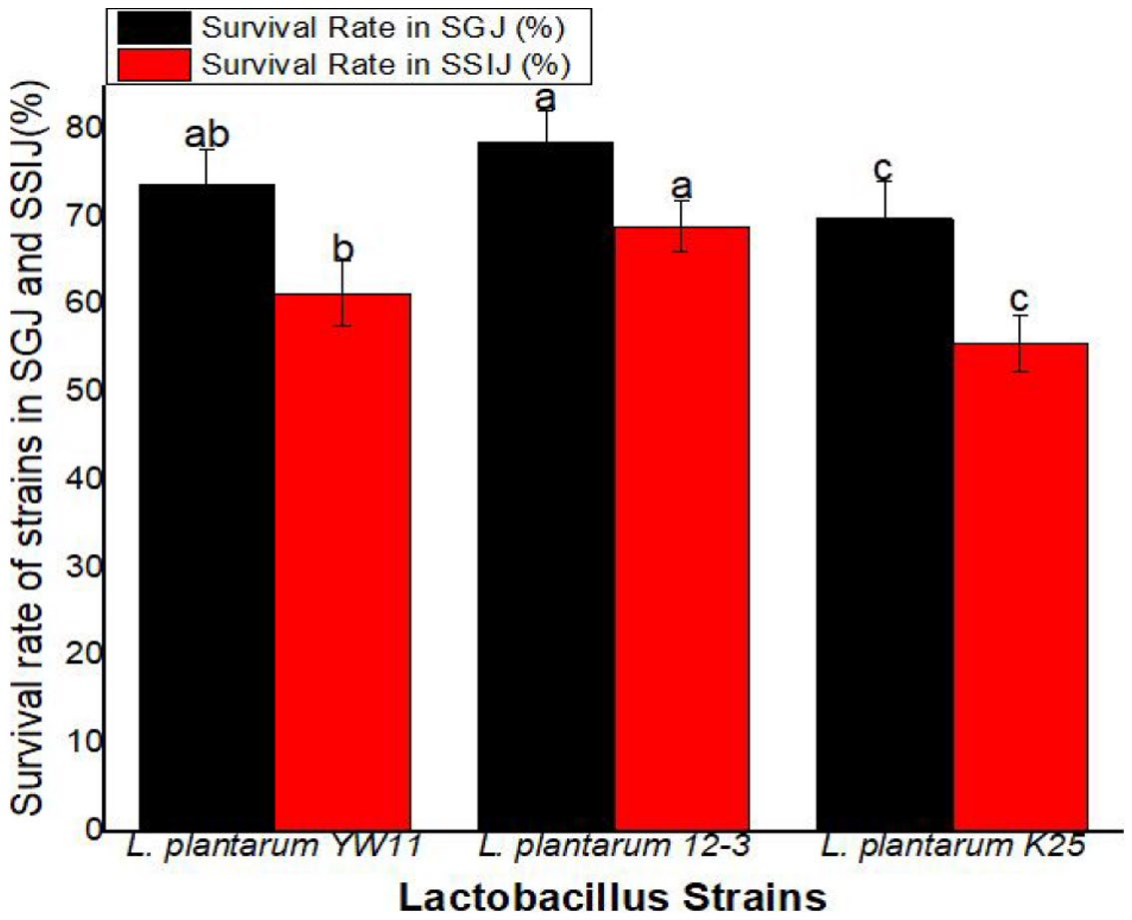
Survival Rate of three strains of *L. plantarum* in Simulated Human Gastrointestinal Transit (SSIJ, simulated small intestinal juice; SGJ, simulated gastric juice). The letters a, b, c showing significant differences (*p* < 0.05) within each column.

The survival rate was greatest for *L. plantarum* 12–3 (78.4 ± 3.6%) followed *L. plantarum* YW11 (73.6 ± 4.1%), and *L. plantarum* K25 (69.8 ± 4.3%) in SGJ. These findings point to *L. plantarum* 12–3 as the strain with the highest resilience to the stomach’s acidic environment. *L. plantarum* 12–3 also had the greatest survival rate in SSIJ, with a mean of 68.9 ± 2.9% while *L. plantarum* YW11 coming in second at 61.2 ± 3.7% and *L. plantarum* K25 in third at 55.6 ± 3.2%. Overall, *L. plantarum* 12–3 showed the best survival rates in both SGJ and SSIJ, showing its capability of colonizing and surviving in the gastrointestinal system. Survival rates were likewise high for *L. plantarum* YW11, whereas they were lower for *L. plantarum* K25 in the simulated gastrointestinal circumstances (Figure 8).

### 3.8. Antioxidant activity

**Figure 9:**
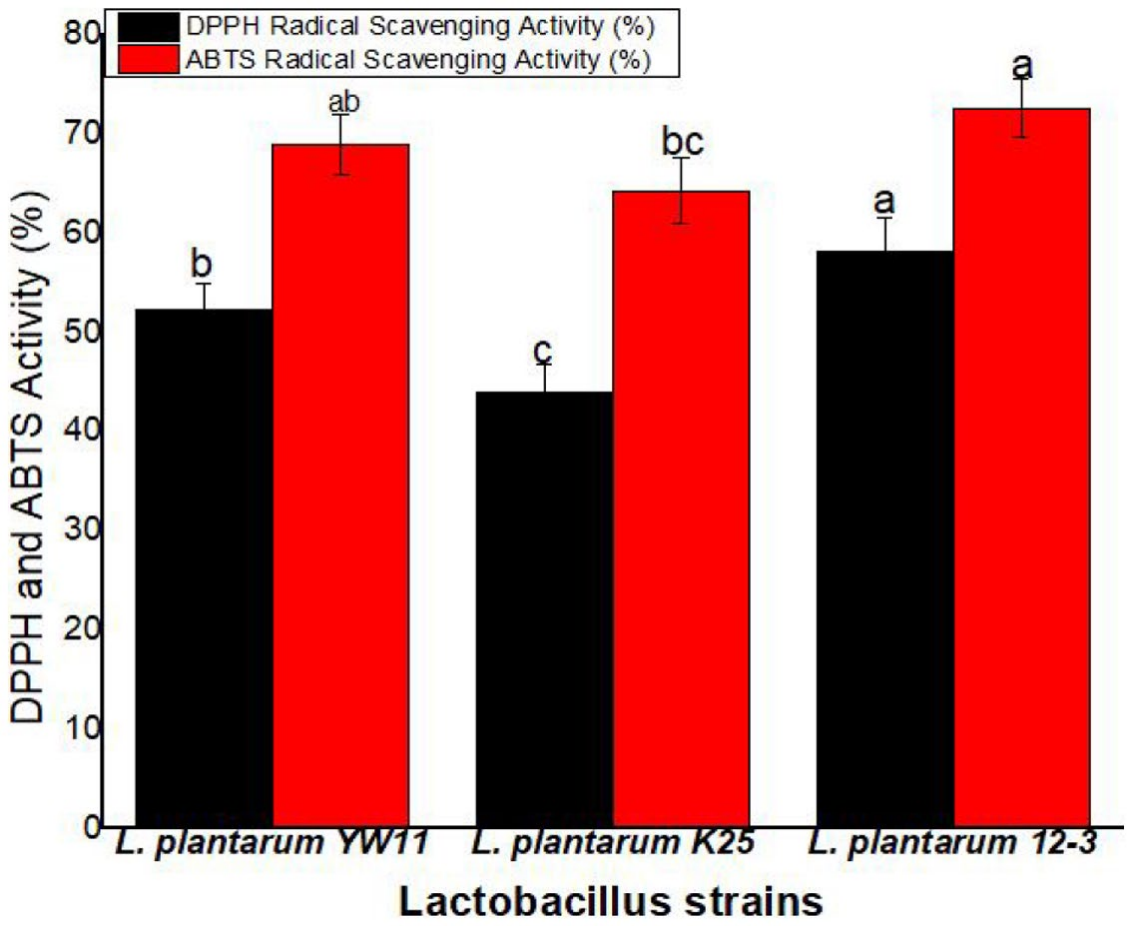
The antioxidant activity of three strains of *L. plantarum* through the DPPH and ABTS radical scavenging tests. The letters a, b, ab, bc, c showing significant differences (*p* < 0.05) within each column.

*Lactiplantibacillus plantarum* 12–3 showed the greatest activity in the DPPH radical scavenging experiment, with a mean scavenging percentage of 58.1% ± 3.4%. The next two most common strains of *L. plantarum* were YW11 (52.3 ± 2.6%) and K25 (43.9 ± 2.6%). According to these findings, *L. plantarum* 12–3 is superior than the other two strains in its ability to scavenge DPPH radicals. *L. plantarum* 12–3 also demonstrated the best activity in the ABTS radical scavenging experiment, with a mean scavenging percentage of 72.6 ± 2.9% (Figure 9).

### 3.9. Antibacterial activity

The antibacterial activity of the three test strain as assessed by diameters of the inhibitory zones developed by each strain against their respective bacteria are listed in Table 3. Some strains had a larger inhibitory zone than others, indicating that they were more effective at killing germs. For examples, the inhibitory zone of *Lactiplantibacillus plantarum* YW11 against *Staphylococcus aureus* was the largest (12.4 mm), followed by those against *Shigella flexneri* (11.2 mm) and *Escherichia coli* (9.7 mm). Inhibitory zones produced by *Lactiplantibacillus plantarum* K25 were 10.4 mm against *Staphylococcus aureus*, 10.7 mm against *Shigella flexneri*, and 8.8 mm against *Escherichia coli*. These results indicate that these *Lactiplantibacillus plantarum* strains have the capacity to limit the development of harmful bacteria, suggesting that they exhibit antibacterial characteristics.

**Table 3:** Antibacterial activity of three *Lactobacillus plantarum* strains.

Strain	Microorganisms	Inhibition	Inhibitory Zone (mm)
*L. plantarum* YW11	*Staphylococcus aureus*	+	10.27 ± 1.07
	*Shigella flexneri*	+	7.36 ± 0.55
	*Escherichia coli*	+	7.0 ± 0.00
*L. plantarum* K25	*Staphylococcus aureus*	+	12.77 ± 1.07
	*Shigella flexneri*	+	7.60 ± 0.36
	*Escherichia coli*	+	7.17 ± 0.29
*L. plantarum* 12–3	*Staphylococcus aureus*	+	11.03 ± 1.40
	*Shigella flexneri*	+	8.07 ± 0.12
	*Escherichia coli*	+	7.67 ± 0.58

Different values along with their standard deviation shows extent of inhibition exhibited by strains, Inhibitory zone in mm, while ‘+” showed they exhibit inhibition and “-“Represents no exhibition.

### 3.10. Hemolytic activity

No hemolytic activity was reported for any of the test strains had any hemolytic activity thus indicating that these strains pose little risk of causing injury or disrupting red blood cells.

### 3.11. Bile salt hydrolase activity

Precipitation circles were produced in the BSH screening media, indicating the strains possess BSH activity. Precipitate circles were generated, which is indicative of BSH activity, however the particular results for the *L. plantarum* strains in this investigation are not provided (data not given).

## 4. Discussion

Probiotics have been studied for their ability to improve digestive health, boost immunity, and lower the risk of diseases and conditions affecting human health. In recent year, the research in investigating potential new sources of probiotic strains and evaluating their valuable qualities is increased several folds. In the current research, we compared the probiotic, antioxidant and antimicrobial effects of oat and soy milk fermented with three distinct strains of *Lactiplantibacillus plantarum*: YW11, 12–3, and K25. Antioxidant activity, tolerance to acids and bile salts, adhesion, and survival in a model of the human digestive tract were also evaluated. The findings indicated that all three strains shown exceptional resistance to the acidic environment and various bile salts and acid concentrations. Consistent with other research, these results show that *Lactiplantibacillus plantarum* strains are highly resistant to gastrointestinal environment. The findings of this study are consistent with those of previous studies that assessed *Lactiplantibacillus plantarum* strains for acid and bile tolerance. Pereira et al. (2003) investigated the acid and bile salt resistance of different *Lactiplantibacillus plantarum* strains and found similar results of enhanced resistance with increasing bile salt concentrations (Pereira et al., 2003). Probiotic strains like *Lactiplantibacillus plantarum* must be able to resist bile salts and acidic conditions in order to thrive and be effective in the gastrointestinal tract. *Lactiplantibacillus plantarum* YW11 was the most resistant to bile salts and acids among the three strains evaluated in our study, with shorter lag phase periods and greater bile resistance percentages. Sun et al. (2022) observed According to the results of the acid resistance experiment, the lag phase all of 44 *Lactiplantibacillus plantarum* strains elevated significantly (*p* < 0.05) when the pH was lowered. While several *Lactiplantibacillus plantarum* strains, particularly *L. plantarum* YW11, displayed increased resistance to bile salts, which is compatible with the current results (Sun et al., 2022).

We further used several different validated assays such as measuring hemolytic activity, bile salt hydrolase (BSH) activity, and antibacterial activity against indicator microbes, to assess the probiotic potential of the test strains. The ability of a potential probiotic to inhibit hemolysis is an important safety indicator. Hemolytically active bacterial strain are not good candidates for probiotics. In the current study, all the test strains of *L. plantarum* including YW11, 12–3, and K25, were found to have no hemolytic activity thus confirming their potential safety as probiotics. Hydrophobicity and auto-aggregation indices were used to compare the adhesion abilities of three *Lactiplantibacillus plantarum* strains (*L. plantarum* YW11, *L. plantarum* 12–3, and *L. plantarum* K25). Hydrophobicity measures the fraction of bacterial cells that interact hydrophobically with surfaces, while auto-aggregation quantifies the cells’ propensity to cluster and form aggregates. This study provides more evidence that adherence to host cells is essential for the probiotic benefits of *Lactiplantibacillus plantarum* strains. Their capacity to adhere to digestive tract surfaces makes them more successful colonizers of the host. These bacteria have demonstrated potential as probiotics due to their hydrophobicity and their ability to self-aggregate. All three strains of *Lactiplantibacillus plantarum* we investigated showed impressive adhesion properties, according to our findings. Our results are comparable with those of previous studies on the adherence abilities of *Lactiplantibacillus plantarum* strains. Different strains of *Lactiplantibacillus plantarum* have been shown to have varying degrees of hydrophobicity and auto-aggregation capacities, as described by Garcia-Gonzalez et al. (2018) in their study of the strains’ adhesion qualities. Their findings suggest that adhesion potentials can vary greatly between strains of *Lactiplantibacillus plantarum*, hence drawing attention to the strain-specific features of this bacterium (Garcia-Gonzalez et al., 2018).

Cholesterol-lowering ability of probiotic strains is another important aspect of probiotics potential and application. In the current study, we used a BSH screening medium similar to compare the BSH activity of the three *Lactiplantibacillus plantarum* strains following standard protocol (Meng et al., 2021). Precipitation rings in the screening media are evidence that the strains are producing BSH. All 44 *Lactiplantibacillus plantarum* strains we investigated formed precipitate rings in our experiments suggesting they are good candidates for probiotics that lower cholesterol. The fact that the inhibitory zone sizes vary across different *Lactiplantibacillus plantarum* strains gives rise to the hypothesis that the efficiency of these strains against various bacteria varies as well. The most inhibitory activity was shown by *L. plantarum* strain YW11, particularly against *Staphylococcus aureus*. In contrast, the inhibitory potential that *L. plantarum* K25 shown against all of the microorganisms that were tested was somewhat lower, and the inhibitory activity that *L. plantarum* 12–3 displayed was intermediate. It’s possible that the antibacterial capability of *L. plantarum* is related to the antibacterial chemicals that are formed by strains, such as antibacterial peptides, hydrogen peroxide, and organic acids. Because of the differences in the widths of the inhibitory zones, it is possible that the strains generate antimicrobial compounds of varying kinds and/or amounts. However, the precise antimicrobial chemicals generated by these strains are yet to be identified, characterized, and their mechanisms of action uncovered in future research. Because *Lactiplantibacillus plantarum* strains have been shown to have antibacterial activity against *Staphylococcus aureus*, *Shigella flexneri*, and *Escherichia coli*, it is possible that these strains might be used as natural agents to inhibit the growth of harmful bacteria.

We further tested the antioxidant properties of oat and soy milk fermented with these *Lactiplantibacillus plantarum* strains in addition to their probiotic potential. Antioxidants are crucial for protecting body against the damaging effect of free radicals. We used two standard assays to measure the antioxidant capabilities of the fermented milk: the 2,2-diphenyl-1-picrylhydrazyl (DPPH) radical scavenging activity assay and the 2,2′-azino-bis(3-ethylbenzthiazoline-6-sulfonic acid) (ABTS) radical cation scavenging activity assay. Two common methods for measuring antioxidant strength are the DPPH and ABTS tests. Our results show that fermented oat and soy milk have strong radical scavenging properties, suggesting they may be useful as a nutritional supplement. Many strains of *Lactiplantibacillus plantarum*, including *L. plantarum* YW11 in particular, have been shown to increase the antioxidant capacity of milk through the fermentation process by producing antioxidant molecules. Benefits to human health from fermented milk’s antioxidant properties are possible. The risk of chronic illnesses including cardiovascular disease, neurological disorders and cancer has been linked to higher consumption of antioxidant-rich foods and drinks. Therefore, the *Lactiplantibacillus plantarum* fermented oat and soy milk have promising use as functional foods due to their high antioxidant potential. In another study, researchers looked specifically at plantaricin K25, a bacteriocin produced by *Lactiplantibacillus plantarum* K25. This bacteriocin was shown to be effective against a wide range of bacteria, both Gram-positive and -negative. Purified plantaricin K25 has a molecular weight of 1772 Da, as measured by MALDI-TOF spectroscopy. The ability of plantaricin K25 to promote cytoplasmic membrane permeabilization and hole formation in susceptible bacterial cells explains its antibacterial effect. These findings suggest that plantaricin K25 is bactericidal because of its action on bacterial membranes (Wen et al., 2016). Another research indicated that adding honey to oat flour aided in the fermentation and storage success of *Limosilactobacillus fermentum* PC1. As a result, the levels of antioxidants, phenolics, and -glucan were all improved. Based on the results of this research, a probiotic meal rich in probiotic and bioactive components may be created by fermenting oat flour with honey and *Limosilactobacillus fermentum* PC1 (Chen et al., 2020).

The therapeutic potential of higher consumption of antioxidant-rich foods and drinks. Therefore, the *Lactiplantibacillus* genotypes has been revealed by research. Mechanistically investigating their resistance to gastric acid and bile salts is an essential property. Important strain-specific properties for specific applications include adhesion ability, antibacterial activity, biofilm formation, and interactions with host cells. Only *in vivo* studies, such as animal models and clinical trials, can reveal the health benefits and efficacy of these strains. Future research should concentrate primarily on maximizing their viability, utility, and health benefits when combined with other food matrixes. Before human consumption is ever contemplated, antibiotic resistance, toxin production, and potential adverse effects must all be exhaustively investigated. Using comparative research, the optimal combinations of Lactobacillus species for various health benefits can be determined.

## 5. Conclusion

In summary, results of the current study points towards substantial probiotic potential of the three test strains. They were able to thrive in the oat and soy milk environments suggesting that they would be able to live in the human digestive system too. The strains have also shown promising activities such as acid and bile salts resistance. In addition the antioxidant activity were enhanced during fermentation, as evidenced by the increased antioxidant capacities of the fermented milk samples. Since no strains showed any hemolytic activity, it was deduced that these bacteria might be safely employed in food fermentation. These results highlight the potential of fermented products as functional foods with enhanced health-promoting qualities by demonstrating the appropriateness of oat and soy milk as substrates for *L. plantarum* fermentation. However, it was shown that the selected strains had varying degrees of probiotic capacity, possibly due to gene expression and regulation changes. To fully understand the effects of these fermented milk products on human health, further study of their fundamental mechanisms is required.

## Data availability statement

The data presented in the study are deposited in the NCBI repository, with the following accession numbers of each strain as under: YW11 (https://www.ncbi.nlm.nih.gov/assembly/GCF_004028295.1); K25 (https://www.ncbi.nlm.nih.gov/assembly/GCA_003020005.1); 12-3 (https://www.ncbi.nlm/nih.gov/assembly/GCF_004028335.1).

## Author contributions

TA: Conceptualization, Writing – original draft, Data curation, Formal analysis. HX: –––. AbS: Conceptualization, Writing – review & editing. MN: Data curation, Methodology, Validation, Writing – review & editing. MAS: Methodology, Writing – review & editing. AK: Data curation, Writing – review & editing. TU: Investigation, Writing – review & editing. MS: Writing – review & editing. YZ: Methodology, Funding acquisition, Supervision, Writing – review & editing. AS: Conceptualization, Writing – review & editing. MYS: Validation, Writing – review & editing. SAA: Investigation, Writing – review & editing. MAT: Investigation, Writing – review & editing. RSJ: Funding acquisition, Writing – review & editing.

## Funding

The author(s) declare financial support was received for the research, authorship, and/or publication of this article. This research work was financially supported by National Natural Science Foundation of China (Project No. 32272296). This work was supported by Princess Nourah bint Abdulrahman University Researchers Supporting Project number (PNURSP2023R31), Princess Nourah bint Abdulrahman University, Riyadh, Saudi Arabia.

## Conflict of interest

The authors declare that the research was conducted in the absence of any commercial or financial relationships that could be construed as a potential conflict of interest.

## Publisher’s note

All claims expressed in this article are solely those of the authors and do not necessarily represent those of their affiliated organizations, or those of the publisher, the editors and the reviewers. Any product that may be evaluated in this article, or claim that may be made by its manufacturer, is not guaranteed or endorsed by the publisher.

## Footnotes

^1^ https://www.ezbiocloud.ne
