# Supplementary material for: Assessing the probiotic potential, antioxidant, and antibacterial activities of oat and soy milk fermented with Lactiplantibacillus plantarum strains isolated from Tibetan Kefir
Source: Front Microbiol. 2023 Sep 25;14:1265188. doi: 10.3389/fmicb.2023.1265188 (PMC10560984; doi:10.3389/fmicb.2023.1265188)
Supplement: Supplementary file 2 [file Table_2.DOCX]

Table 1 The antibacterial activity of *Lactobacillus plantarum*

| Indicator microorganism | Staphylococcus aureus | Escherichia  coli | Shigella  flexneri | Enterobacter sakazakii |
| --- | --- | --- | --- | --- |
| YW11 | 10.27±1.07 | 7.00±0.00 | 7.36±0.55 | 9.60±0.26 |
| K25 | 12.77±1.07 | 7.17±0.29 | 7.60±0.36 | 9.63±1.32 |
| 12-3 | 11.03±1.40 | 7.67±0.58 | 8.07±0.12 | 13.63±1.96 |


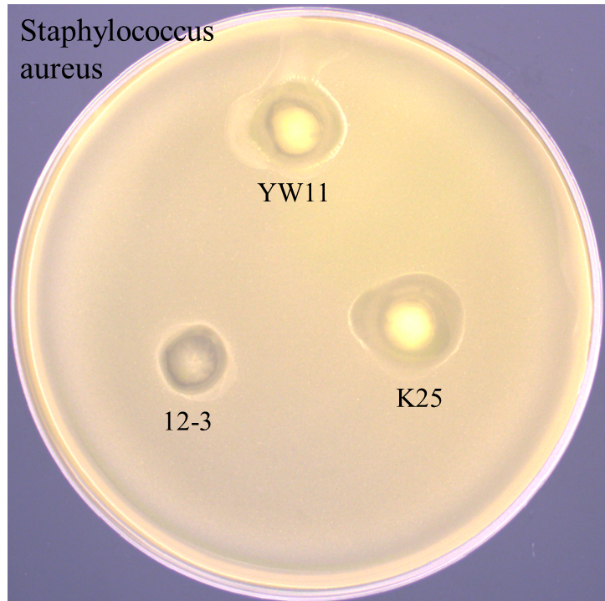

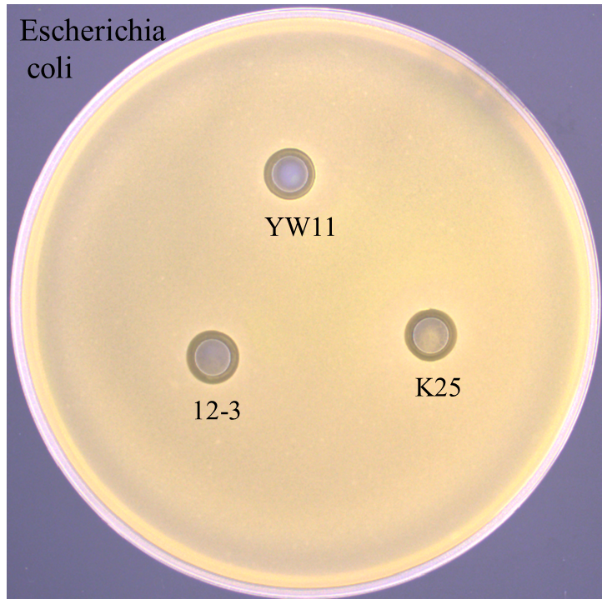


##
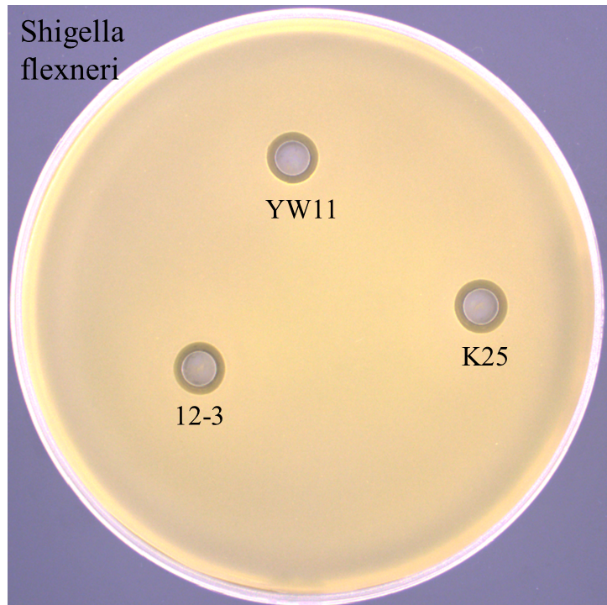

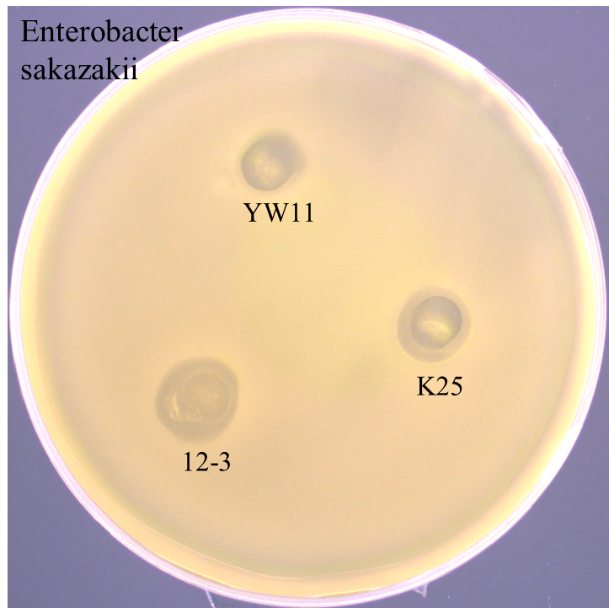


## Supplementary Figure1: Antibacterial resistance of lactobacillus strains against commonly pathogen.

**
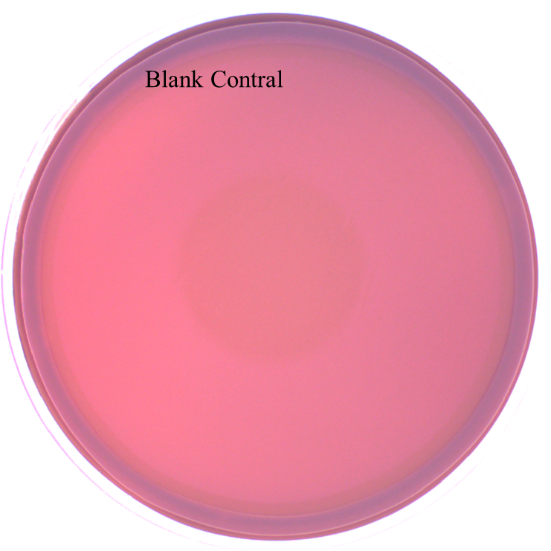

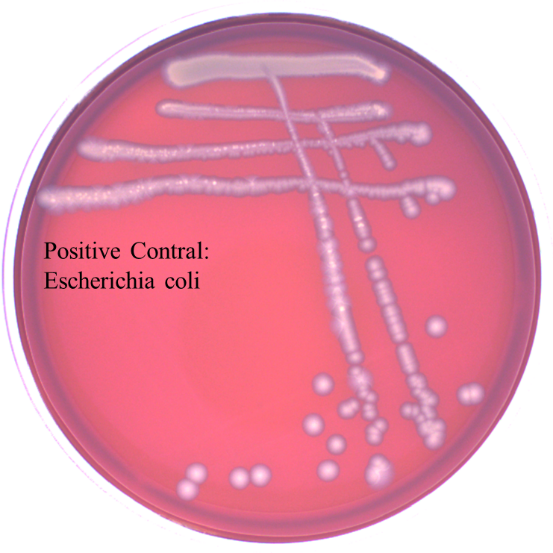
**

**
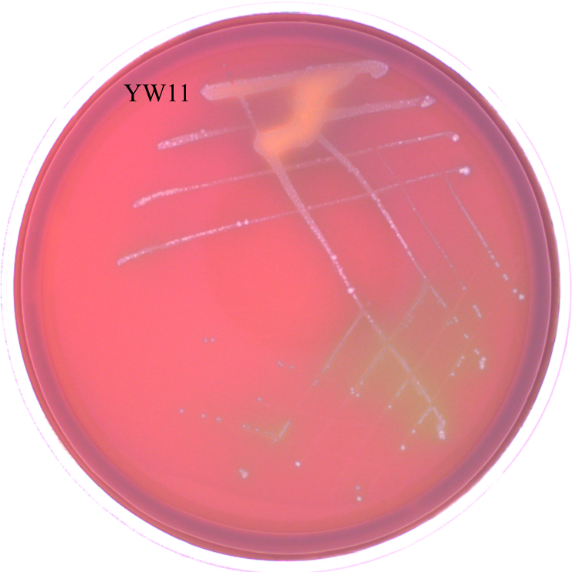

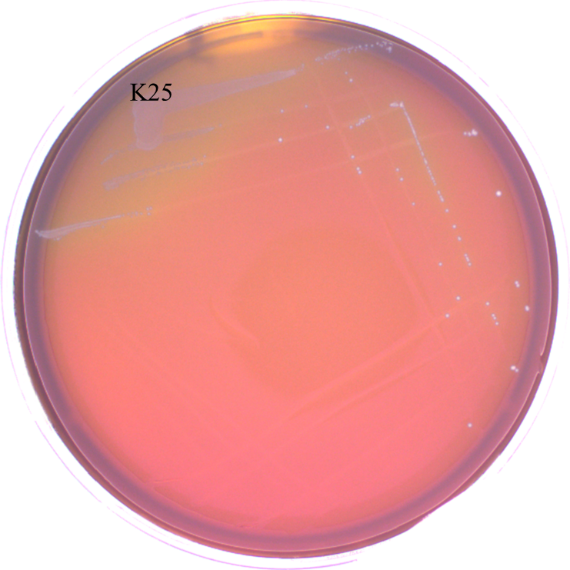
**

**
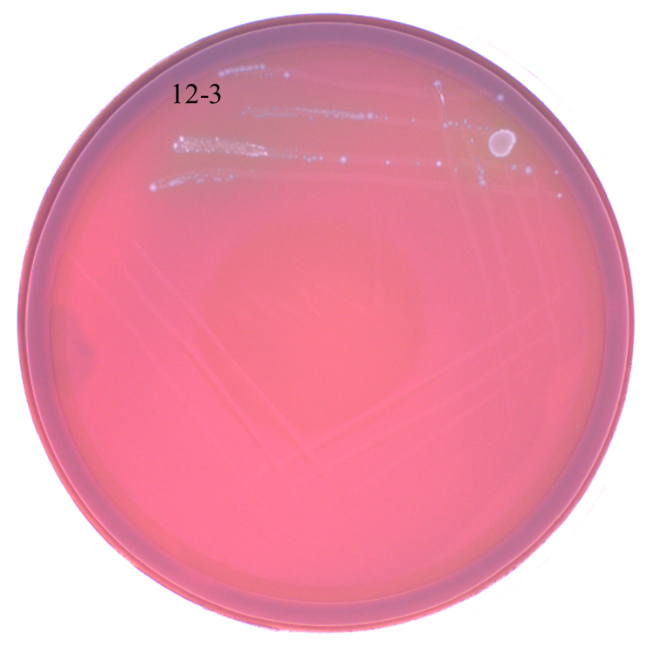
**

**Supplementary Figure2: Hemolytic activity of Lactobacillus strains**
